# Alternatives for nitrate and nitrite in fermented meat products: potential contribution of the nitric oxide synthase activity of coagulase-negative staphylococci

**DOI:** 10.1186/2049-3258-72-S1-O4

**Published:** 2014-06-06

**Authors:** María Sánchez Mainar, Stefan Weckx, Luc De Vuyst, Frédéric Leroy

**Affiliations:** 1 Research Group of Industrial Microbiology and Food Biotechnology, Faculty of Sciences and Bioengineering Sciences, Vrije Universiteit Brussel, B-1050 Brussels, Belgium

## Background

Nitrosomyoglobin, which is the cured colour of fermented meat products, results from the interaction between muscle-based myoglobin and nitric oxide (NO) [1]. NO originates from the addition of nitrate and/or nitrite as curing agents to the meat batter. During fermentation, nitrate is reduced into NO-yielding nitrite by coagulase-negative staphylococci (CNS), present in the meat or added as starter culture [2]. However, health concerns related to the consumption of cured meats are leading to research for alternatives to generate the cured colour. A yet poorly explored pathway could potentially be based on the action of nitric oxide synthase (NOS), which produces NO from arginine. Bacterial NOS activity has only been scantily described, particularly its potential presence in meat-related bacteria and its dependency on environmental conditions. Based on preliminary attempts [3], and because up to now none of the sequenced *Lactobacillus* species contain a NOS homologue [4], this study focused on meat-related CNS.

## Materials and methods

A genotypic screening for the presence of the NOS-encoding gene and a phenotypic screening for the conversion of arginine via NOS activity and other alternative pathways metabolising arginine were performed for 88 CNS strains. Also a complementary screening for potential NOS-stimulating conditions and a kinetic analysis of possible NOS activities in CNS were done in laboratory fermentors and meat models.

## Results

The genetic potential for NOS activity was frequently found among CNS strains. The phenotypic screening confirmed that arginine metabolism was common, which resulted in mixtures of citrulline and mostly ornithine, with considerable variability on species and strain level, indicative of arginine deiminase activity. The production of citrulline without ornithine formation, indicative of potential NOS activity, was not found under the conditions tested, except for the strain *S. haemolyticus* G110. However, kinetic experiments indicated that *S. haemolyticus* G110 was not able to demonstrate NO-driven colour formation in fermented meats, highlighting the importance of technological adaptation of functional candidate strains. Attempts to express the *nos* gene in other CNS strains were unsuccessful, suggesting that the genetic potential is not commonly expressed by CNS.

## Conclusions

The use of NOS-positive bacterial cultures for nitrate and nitrite cutback in fermented meats is not straightforward. A bottleneck seems to be on the gene expression level, whereas phenotypically positive strains also need to be technologically adapted to the meat fermentation process.

## References

[B1] Ordóñez J Hierro E Bruna J De la Hoz L Changes in the components of dry fermented sausages during ripening Crit Rev Food Sci Nutr 1999 39 329 367 10.1080/10408699991279204 10442271

[B2] Leroy F Verluyten J De Vuyst L Functional meat starter cultures for improved sausage fermentation Int J Food Microbiol 2006 106 270 285 10.1016/j.ijfoodmicro.2005.06.027 16213053

[B3] Morita H Sakata R Nagata Y Nitric oxide complex of iron (II) myoglobin converted from metmyoglobin by Staphylococcus xylosus J Food Sci 1998 63 352 355

[B4] Crane B Sudhamsu J Patel B Bacterial nitric oxide synthases Annu Rev Biochem 2010 79 445 470 10.1146/annurev-biochem-062608-103436 20370423

